# Global priority for the care of orphans and other vulnerable children: transcending problem definition challenges

**DOI:** 10.1186/s12992-023-00975-0

**Published:** 2023-10-10

**Authors:** Yusra Ribhi Shawar, Jeremy Shiffman

**Affiliations:** 1 Johns Hopkins University, Bloomberg School of Public Health, Baltimore, MD USA; 2 Johns Hopkins University, Paul H. Nitze School of Advanced International Studies, Washington, D.C, USA

**Keywords:** Children’s care, Child wellbeing, Orphan, Institution, Deinstitutionalization, Alternative care

## Abstract

**Background:**

Tens of millions of children lack adequate care, many having been separated from or lost one or both parents. Despite the problem’s severity and its impact on a child’s lifelong health and wellbeing, the care of vulnerable children—which includes strengthening the care of children within families, preventing unnecessary family separation, and ensuring quality care alternatives when reunification with the biological parents is not possible or appropriate—is a low global priority. This analysis investigates factors shaping the inadequate global prioritization of the care of vulnerable children. Specifically, the analysis focuses on factors internal to the global policy community addressing children’s care, including how they understand, govern, and communicate the problem.

**Methods:**

Drawing on agenda setting scholarship, we triangulated among several sources of data, including 32 interviews with experts, as well as documents including peer-reviewed literature and organizational reports. We undertook a thematic analysis of the data, using these to create a historical narrative on efforts to address children’s care, and specifically childcare reform.

**Results:**

Divisive disagreements on the definition and legitimacy of deinstitutionalization—a care reform strategy that replaces institution-based care with family-based care—may be hindering priority for children’s care. Multiple factors have shaped these disagreements: a contradictory evidence base on the scope of the problem and solutions, divergent experiences between former Soviet bloc and other countries, socio-cultural and legal challenges in introducing formal alternative care arrangements, commercial interests that perpetuate support for residential facilities, as well as the sometimes conflicting views of impacted children, families, and the disability community. These disagreements have led to considerable governance and positioning difficulties, which have complicated efforts to coordinate initiatives, precluded the emergence of leadership that proponents universally trust, hampered the engagement of potential allies, and challenged efforts to secure funding and convince policymakers to act.

**Conclusion:**

In order to potentially become a more potent force for advancing global priority, children’s care proponents within international organizations, donor agencies, and non-governmental agencies working across countries will need to better manage their disagreements around deinstitutionalization as a care reform strategy.

**Supplementary Information:**

The online version contains supplementary material available at 10.1186/s12992-023-00975-0.

## Background

Every day, families are disrupted across the world due to parental death, incapacity, abuse, and/or neglect. The result is millions of children without parental care. By one estimate, there are 147 million orphans [1]—children under 18 years of age who have lost one or both parents to any cause of death—and millions more across the world separated or at risk of separation. COVID-19 exacerbated the problem [2–4]. The absence of quality care results in bleak long-term physical health, psychological, and social outcomes for children [5].

Especially over the last decade, various bilateral donors, foundations, multilateral agencies, non-governmental organizations, faith-based actors and consortiums have increased efforts to address the care of these at-risk children—an issue that intersects health, protection, social welfare and education sectors. Understood as the children’s care agenda, it includes strengthening the care of children within families, preventing unnecessary family separation, and ensuring quality care alternatives when reunification with biological parents is not possible or appropriate. An increasingly dominant aspect of the children’s care agenda is reform of the children’s care system [6]. A children’s care system refers to the actors and processes aimed at providing services for vulnerable families to prevent unnecessary separation, and at offering alternative care (any arrangement, formal or informal, temporary or permanent, such as kinship, community-based, residential-based care) for children who cannot be cared for in their biological families.

Despite the growing prominence of children’s rights and wellbeing discourses [6–10] children’s care remains inadequately prioritized globally. There is little mention of the care of orphans and other vulnerable children in major goals and resolutions, including the Sustainable Development Goals (SDGs); there are few government resource commitments to and inadequate implementation of the United Nations Guidelines for the Alternative Care of Children, especially given inadequacies in the social welfare workforce and systems in many countries; [11, 12] many countries still lack policies and clear governance frameworks that regulate and oversee cohesive, appropriate and quality care arrangements for orphaned and other vulnerable children [13], and where organizational documents concerning the care of vulnerable children exist, they are typically subsumed in discussions of violence against children. The lack of global priority is also reflected in insufficient donor funding for the issue [14].

While there is growing literature examining the determinants of and potential solutions for improving children’s care and the well-being of orphans, there is comparatively little knowledge about what shapes global political priority for care of these children. We analyze the factors shaping the inadequate global prioritization of the care of vulnerable children. While multiple factors stand behind low global priority for children’s care, including the limited power of affected children and families, the issue’s multi-sectoral nature, and competition for attention with other social welfare issues, our analysis focuses on factors internal to a global policy community addressing children’s care, referred to as children’s care proponents. An examination of the internal dynamics of proponents concerned with children’s care is critical given that much of the existing literature [15–18] assumes this community understands children’s care, childcare reform, and specifically deinstitutionalization (DI) in the same way. A clear understanding of these factors is essential for proponents concerned with children’s care to identify better strategies to augment priority for childcare reform, as well as the broader children’s care agenda.

### Childcare reform and deinstitutionalization of orphans

The children’s care agenda includes at least three components: (1) strengthening the ability of families to care for their children, (2) preventing family separation in groups most at risk, and (3) providing suitable and appropriate alternative care for the millions of children separated from their biological parents [19, 20]. The latter component concerns child care reform, with the strategy of DI most recently being core to this. DI refers to the process of reforming childcare systems by closing down orphanages and institutions, preventing the opening of new ones, and replacing institutional care for children with care in a family or family-like environment in the community [21–23]. Historically, institutionalization of orphans and vulnerable children was a common practice across Europe. Foundling homes were first established in Italy in the fourteenth century in response to the growing number of abandoned babies, a practice that then spread to other parts of Europe and Russia [24]. Orphanages also appeared across the region, and subsequently in many LMICs given colonial influences [25]. Drivers of institutionalization included poverty, social deprivation and poor parenting skills, child illness and disability, natural and human-made disasters, and child abuse and neglect [26].

Since the 1970s, DI of orphans and vulnerable children became the policy and practice orthodoxy across Western Europe and North America, Australia and New Zealand; this was largely linked to the professionalization of child welfare and the emergence of child protection agencies [27, 28]. The DI discourse began to emerge in Romania and other Eastern European countries in the 1990s, after horrifying images surfaced of thousands of neglected children housed in overcrowded, state-run orphanages [29]. These images sparked public outrage and brought attention to the developmental delays and abnormal behavior in children resulting from institutionalization.

Since then, and especially in the last decade, there has been increasing commitment to advance DI among international organizations and donors across the world. This is reflected in the development of international commitments, such as the 2010 United Nations Guidelines for the Alternative Care of Children, 2019 United Nations General Assembly Resolution on the Rights of the Child, 2021 Committee on the Right of the Child Day of General Discussion on Children’s Rights and Alternative Care, and 2022 Guidelines on Deinstitutionalization [6–9]. It is also reflected in regional efforts in Europe, Latin America, and Asia [30–32]. However, in many parts of the world, the ‘institution’—broadly defined to encompass orphanages, large-scale institutions, and sometimes, small group homes and children’s villages—continues to be used as the main form of alternative care. It is estimated that approximately 5.37 million children between the ages of 0 and 17 years could be living in institutional care worldwide [18].

### Theory on agenda-setting

Political science and sociological research on collective action has identified factors that shape the level of public and governmental attention that societal problems, such as children’s care, receive. Specifically, we draw on policy frameworks grounded in theory on collective action that examine political priority of global health issues and the role that global health networks play [33, 34]. These frameworks, which draw on broader social science literature, have been applied to understand various health and social issues including violence against children, early childhood development, and gender equity [35–37]. They identify three key categories of factors that shape the level of global political priority an issue receives: issue characteristics—inherent features of the problem itself; the policy environment—the political developments, structures, and social norms that surround the issue, and the nature of proponents—the characteristics of and strategies employed by individuals and organizations concerned with the problem.

With respect to issue characteristics, issues are more likely to garner attention when they affect groups that societies view sympathetically and have significant political power, as well as when they are perceived to be relatively simple to address [38–40]. In terms of policy environment, issues are more likely to garner priority when norms, institutions, and funding for the issue favorably align, and when policy windows open [41]. With respect to the nature of proponents and the strategies they employ, research reveals the central influence of *problem definition—*a social process involving the identification of the causes, consequences of and solutions to a problem [42, 43]. Problem definition shapes how policy-makers think and talk about particular concerns, and affects the rise of issues on policy agendas [44]. Organizations are more likely to act on a problem when proponents come to evidence-based agreements on what the problem is, how it should be addressed, and why it is important. Action may be hampered when proponents become embroiled in conflict on the nature of the problem [45, 46].

Effectiveness in defining the problem shapes two other challenges proponents commonly face: *governance*—the creation of institutions to bring about collective action and advance coalition-building, and *positioning*—the portrayal of the issue in ways that inspire external audiences to act [47]. With respect to governance, problem definition disagreements, when ineffectively managed, may lead to difficulties in identifying leaders and setting up institutions capable of guiding collective action, and in forging alliances with external actors whose support and resources may be necessary to advance the issue [48]. Challenges with problem definition may also lead to positioning problems—difficulties with framing the issue in ways that attract the support of political leaders, particularly if proponents are unable to develop a coherent ‘ask’ of these leaders and portray the issue in ways that resonate with their values and interests [49, 50].

Research on advocacy coalitions suggest that generating consensus within policy communities can be difficult, but that disagreements may be managed productively without fragmenting a community. One means is through policy-oriented learning, a gradual accumulation of information through scientific studies and new stakeholder experiences [51]. Another mechanism is via a hurting stalemate: contending parties come to view a continuation of the status quo as unacceptable, and become more willing to compromise. Consensus may also emerge through processes external to policy communities such as external shocks that force communities to reconsider strategy and take immediate action [52].

Drawing on agenda setting scholarship and triangulating data from interviews with experts in children’s care and a literature review, we investigate what has hindered children care’s global policy advancement, especially as it concerns factors that are internal to the global policy community addressing children’s care, including how they understand, govern, and communicate the problem.

## Methods

### Data

In order to identify the factors potentially shaping inadequate global prioritization of children’s care, we triangulated among several sources of data, including interviews with key informants, as well as documents including peer-reviewed literature, organizational reports and media.

#### Literature review

We searched the Google Scholar database and websites of organizations concerned with children’s care. The search terms used were: “children”, “child”, and/or “orphan”, in combination with “care”, “care reform”, “alternative care”, “orphanage”, “institutions”, “informal care”, “community-based care”, “family-based care” “foster care”, “adoption”, “kinship care”, and/or “deinstitutionalization”. The search was restricted to literature in English, between the years 1960 and 2022 to capture the time period in which the strategy shifted from institutionalization of orphans and other vulnerable children to DI. We also restricted our search to documents that pertained to the strategies, arguments, and policies that global children’s care actors have considered in improving the care and well-being of orphans and other vulnerable children and advancing the issue globally. We also included policy and programmatic challenges pertaining to children’s care and the historical evolution of child care reform across various countries and regions, especially in low and middle-income countries in South and East Asia, Eastern Europe and Sub-Saharan Africa. Given the aims of our study, we excluded documents that focused on the prevalence of orphans and vulnerable children in various forms of care, evaluation of program interventions related to children’s care, and articles that were focused at the sub-national level or exclusively focused on one country, without discussion of its influence on global-level actors or dynamics. We conducted the literature review prior to the semi-structured interviews with children’s care actors to identify emergent themes around challenges and opportunities in advancing children’s care on the global agenda, support the development of the key informant interview guide, and identify potential key informants for interview. We also conducted a second literature review following the completion of interviews to include key documents that were noted by key informants, as well as grey literature, including strategy and policy documents from key global actors in children’s care (e.g., UN agencies, the Better Care Network, Lumos, Foundation Changing the Way We Care, and SOS Children’s Villages International). In total 182 documents were included and reviewed.

#### Key informant interviews

In addition, we conducted 32 semi-structured interviews with actors central to child care advocacy, research, and/or programming across multiple countries, as well as observers of global child care efforts actors who are more generally concerned with child well-being (see Table 1 for organizational affiliations). Employing a purposive rather than representative sampling strategy, we identified these individuals through our initial literature review and by asking respondents whom they considered to be most centrally involved in children's care programming, research and/or advocacy. 34% of the respondents were from international non-governmental organizations; 22% from networks or alliances dedicated to children’s care or well-being; 19% from foundations; 9% from academic institutions; 9% from consulting companies; and 6% were from international organizations. 36 individuals were contacted for an interview (88% response rate). Four individuals did not respond to our invitation for interview; their non-response is unlikely to have affected the study’s results given the overall low non-response rate and our ability to secure interviews with other individuals working within the same organization. The interviews took place between November 2018 and June 2021, lasting on average one hour and twenty minutes and all were conducted over Skype or telephone. We continued to interview key informants until we reached theoretical saturation—the point at which we obtained no new critical information from additional interviews [53]. The interview questions were open-ended and tailored to each individual’s background, although some questions posed were consistent across all those interviewed (see Annex 1 for the template of questions used).

**Table 1:** Organizational affiliation of key informants

ACC International	Lumos Foundation
Better Care Network	Maestral International
Better Volunteering, Better Care	Migration Policy Institute
Catholic Relief Services	Miracle Foundation
Changing the Way We Care	New York State Department of Social Services
Child Frontiers	Oak Foundation
Children and Youth Economic Strengthening Network	Rethink Orphanages
Defence for Children International	Save the Children
Disability Rights International	SOS Children’s Villages International
Doris Duke Foundation	United Nations Children's Fund (UNICEF)
Duke University	University of Central Lancashire
Elevate Children Funders Group (includes among other organizations: GHR Foundation, Oak Foundation, World Childhood Foundation)	University of Chicago
Faith to Action	United States Agency for International Development (USAID)
Family for Every Child	Washington Network for Children and Armed Conflict
GHR Foundation	Whole Child International
Hope and Homes for Children	World Childhood Foundation
International Social Service (ISS)	

### Analysis

Adhering to the Consolidated Criteria for Reporting Qualitative Research (COREQ), [54] we employed a qualitative case study and undertook a thematic analysis [55] of the collected documents and interview transcripts, using these to create a historical narrative on efforts to address children’s care, and specifically childcare reform. Thematic analysis is a method for identifying and understanding patterns of experiences and meaning and is useful in examining the social processes that shape particular phenomena, such as policy change [56]. The focus of this analysis is at the global level—the actors and processes that span transnational boundaries—rather than specific national or grassroots actors and debates, except in instances in which national dynamics had influenced or been influenced by global children’s care advocacy efforts. We take no position on the policy debates identified; our aim rather is to explore how members of the children’s care policy community understand DI and the effects of their differences on global priority for children’s care.

#### Code development and data extraction

We used an iterative process in developing the codes [55]. The first author originally coded data from the literature review and key informant interviews by two broad categories derived from policy frameworks that examine the determinants of political priority for global health and social development issues [33, 34]. These categories are (1) the characteristics of the issue—understood as the features inherent to the problem, such as its prevalence, causes, drivers, and impacts on particular populations; and (2) the internal dynamics of the involved actors—understood as the strategies, decision-making processes, and actions of those actors concerned with the well-being and care of orphans and other vulnerable children.

The coding evolved as additional data were collected. Additional sub-codes evolved based on additional ideas from scholarship on policy process and particularly problem definition, [42, 44–46, 57] as well as empirical data relevant to understanding challenges or opportunities to advancing children’s care on the global agenda. For example, under the issue characteristics parent code, the following sub-codes were created: data contradictions on prevalence and nature of problem, insufficient evidence on effective solutions, divergent experiences across regions, socio-cultural norms around family and care, capacities of national social protection and legal systems, and commercial interests. Under the internal dynamics of involved actors parent code, the following sub-codes were created: problem definition (with further sub-codes created for each of the distinctive positions on DI that we identified: pro-DI, progressive-DI, and DI-critical), governance (further sub-codes created around leadership, coordination, and coalition-building), and positioning (further sub-codes created around ambiguous terminology, orphan misconceptions, DI-emphasis).

#### Data validity, reliability and confidentiality

To minimize bias and validate findings, we triangulated among data sources, always corroborating and comparing information from interviews with written sources, rather than relying predominantly on one or the other source of information. In reporting the interview data, we assigned each key informant a number, and listed their most prominent institutional affiliation type and country classification (see Table 2). To ensure historical accuracy and data validity, we incorporated feedback on a draft of this paper from four interviewees representing distinct perspectives. We also asked reviewers to identify any additional documents we should be reviewing that we had overlooked, and none indicated any. To enhance data reliability, interview audio was transcribed and the first author, who undertook all key informant interviews, conducted coding and analysis of the data collected. Both authors are outsiders to the children’s care community—social science researchers who initiated the research without any strong stance on any of the major global policy debates concerning children’s care.

The study protocol underwent ethics review and received exemption by the Institutional Review Board of Johns Hopkins University. All interviews were recorded and transcribed with consent from participants. To ensure respondent privacy and confidentiality, we assigned each respondent a unique number (i.e., I(interview)1, I2, etc.) in coding the data and presenting the results in the manuscript so that they could not be linked to identifying information. We also ensured that all data collected (notes taken during the interview, interview audio, and transcribed interview) and documents outlining data collection approaches were stored in an encrypted, password-protected folder only accessible to authors of this manuscript.

**Table 2:** Key informant number/organizational type

1	International Organization	17	Network/Coalition/Alliance
2	Network/Coalition/Alliance	18	Foundation
3	International NGO	19	International NGO
4	International NGO	20	Academia
5	International NGO	21	Foundation
6	Network/Coalition/Alliance	22	Academia
7	Consulting Company	23	International NGO
8	Network/Coalition/Alliance	24	Foundation
9	International NGO	25	Foundation
10	International NGO	26	Foundation
11	Foundation	27	International NGO
12	International Organization	28	International NGO
13	International NGO	29	Network/Coalition/Alliance
14	Consulting Company	30	Consulting Company
15	International NGO	31	Network/Coalition/Alliance
16	Academia	32	Network/Coalition/Alliance

## Results

**Fig. 1:**
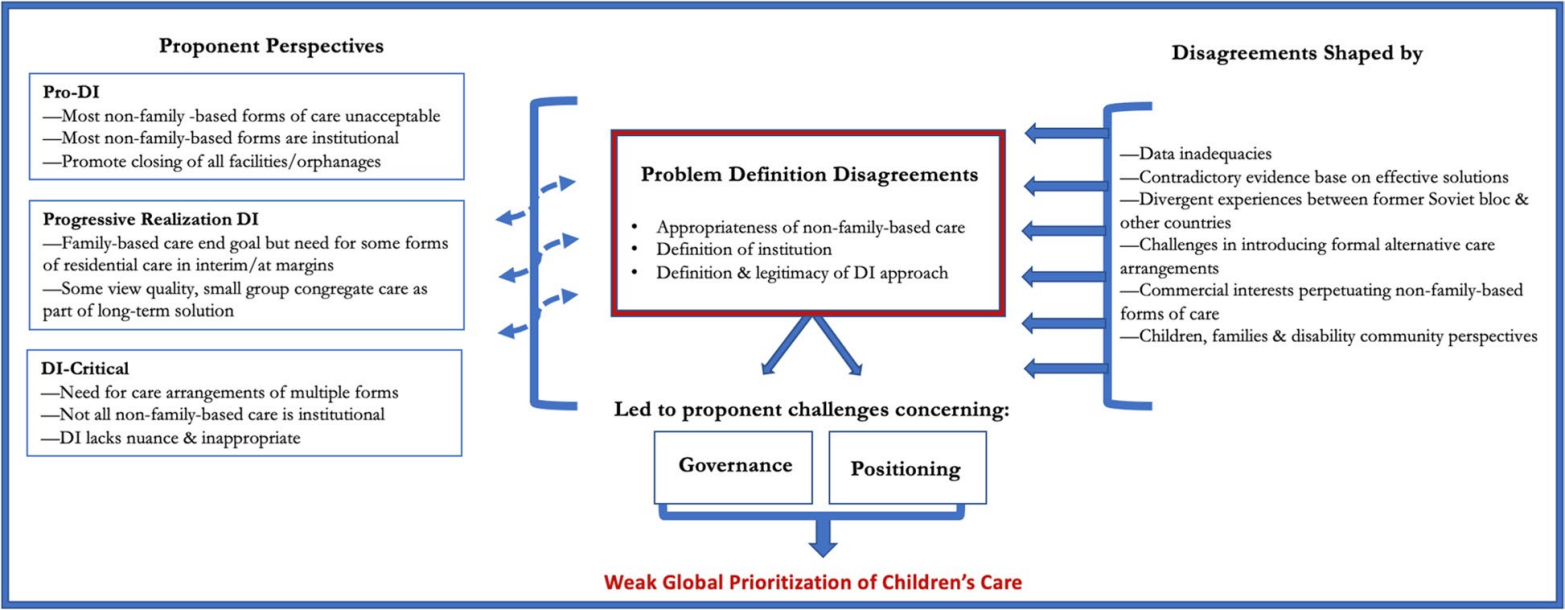
Problem definition disagreements, factors shaping perspective differences & their impact on proponent efforts to advance children’s care

We sought to identify the key factors shaping global priority for children’s care, especially those internal to the community of children’s care proponents. Problem definition disagreements surrounding DI among children’s care proponents is a central challenge to advancing global priority for children’s care. We first discuss this challenge, identifying—through analysis of the data—three distinct perspectives: pro-DI, progressive realization DI, and DI-critical. These distinct perspectives differed in their definition of an institution, what DI encompasses, and the strategy’s legitimacy. We then identify the factors that have shaped children’s care proponent disagreements. We end by identifying two consequences of problem definition disagreement: ineffective governance and unconvincing framing of the problem, both of which have further hampered global advancement of addressing children’s care. Figure 1 summarizes the core beliefs held by three groups of children’s care proponents, the factors shaping perspective differences, and the impact of these divisions on proponent efforts to advance global attention.

### Problem definition disagreement

Findings reveal divisive disagreements among children’s care proponents surrounding deinstitutionalization (hereafter referred to as DI) as a strategy, which are underpinned by disagreements on what DI encompasses, the appropriateness of various forms of non-family-based care, and which forms ought to be labeled ‘institutional’ in nature—a term with negative connotations. Children’s care proponents fall into three groups on the issue of DI, which we term pro-DI, progressive realization DI, and DI-critical. Pro-DI proponents view nearly all non-family-based forms of care to be unacceptable, consider most of these forms to be institutional in nature, and call for the closure of even small-scale facilities and orphanages. Respondents identify Disability Rights International as the organization most aligned with this perspective (I1, I10, I13, I14, I24, I25, I29, I30). Others supporting DI call for a progressive realization approach—with children in families and family-based care as the end goal, but recognizing the need for some forms of residential care in the interim and/or at the margins of the continuum of care. Some view quality, small group congregate care as part of the long-term solution (I1, I10, I14, I29, I30). Those supporting progressive realization include Better Care Network, Lumos Foundation, Hope and Homes, USAID, and the Lancet Institutional Care Reform Commission Group. DI critics (themselves holding varying views concerning what ‘DI’ refers to) view the strategy as lacking nuance and therefore inappropriate, and perceive the need for continuing care arrangements of multiple forms. They see the need for ongoing non-family-based care arrangements when other alternatives are not available, and do not designate all these arrangements as institutional in nature. Respondents identify SOS Children’s Villages International, Whole Child International, Family for Every Child and a small group of academics as embracing this perspective (I1, I7, I8, I10, I14, I18, I20, I25, I27). The organizational histories and locales of key informants informed their adoption of these positions. For instance those working in organizations focused on former Soviet Union states were more likely to have embraced DI, as well as disability organizations given neglect of that group of children and their placement into facilities. In contrast, individuals working in organizations more focused on other parts of the world are less wedded to DI, as they are likely to have observed a diversity of circumstances of children.

Despite differences, nearly all care proponents agree that family-based care is ideal and that very large residential facilities are poor options and should be closed. Momentum to address these differences has accelerated, in part due to children’s care problems exacerbated by the COVID-19 pandemic (I31). A Lancet Commission on Children’s Care helped bridge disagreements, [26] as did a 2019 United Nations General Assembly Resolution on the Rights of the Child, which contained provisions on children without parental care (I3, I14, I29, I30) [58]. In the latter, a coalition of 256 organizations working on children’s care agreed on the need to strengthen children’s care in families, prevent unnecessary separation by addressing its root causes, tackle orphanage volunteering, and put an end to institutionalization by progressively replacing it with family and community-based care. Yet proponents note this agreement did not bridge all differences. One commented:

Many of us in the middle were very frustrated…There is a coming together of minds, but you are not hearing it in these debates (I30).

Another respondent points to the effects of ongoing divisions:

We are bogged down on these technical issues and as a result we’re unable to actually think strategically about…the fundamental issue of how we ensure care for children (I29).

### Differences on the definition of ‘institution’

Differences concerning what constitutes institutional care shape childcare reform debates. The UN Guidelines for the Alternative Care of Children [6] distinguish between ‘institutions’—a term that appears only once in the document—and residential facilities. Residential facilities encompass all alternative care settings that are not family-based (including those that are categorized as ‘family-like’— another term that provokes disagreement) from emergency shelters and small group homes to the biggest residential facilities. The term ‘institutions’ is often reserved only for large residential facilities. However, there is no universally agreed understanding—in the Guidelines or among care proponents—of what constitutes an ‘institution’ as opposed to other kinds of residential care settings, and the terms are used inconsistently among care sector proponents (I3, I9, I25, I27).

These definitional differences have shaped proponent disagreements surrounding which residential care arrangements are institutional in nature and therefore an unacceptable option for children’s care (I2, I5, I7, I8-I10). These include emergency shelters, children’s homes, small group homes, and children’s villages. Those holding pro-DI perspectives find any care arrangements that are not family-based to be institutional in nature (I1, I12, I23, I24):

They are just mini-institutions and no child should be in a small group home. Every child should be in a family (I1).

In contrast, those critical of DI generally consider some arrangements that are not family-based care to constitute residential or family-like care approaches [17, 59]. One respondent noted:

Residential care does not by definition have to be institutional…Most people when they think of residential care they think of the Romanian orphanages. That of course is terrible…and there is no reason for that. The same structure can be restructured to feel like a home (I15).

### Differences on the definition and legitimacy of the DI approach

These disagreements about what constitutes an institution underpin the most divisive debate: whether DI is an appropriate entry point for care reforms, and what DI actually encompasses.

#### Arguments by those who identify as pro or progressive realization ‘DI’

Pro-DI proponents believe that facilities that institutionalize children are unable to guarantee the well-being of children and deprive children of their right to a family. In advancing their perspective, proponents make several arguments.

Pro-DI proponents argue that facilities that institutionalize children have long-term harmful effects on child well-being, and often apply the findings to all forms of care that are not family-based (I1, I3, I4, I12, I21,I23). The literature that supports this claim is sizeable and dates back to the mid-20th century. Studies have found that institutionalized children, in comparison to their peers, are atypically short, low IQ, and low self-esteem [60–62]. Many DI proponents point to the Bucharest Early Intervention Project (BEIP), [63] a randomized-controlled trial comparing longitudinal outcomes among young institutionalized children, which offered strong evidence that institutional care has a causal effect on developmental deficits and delays. This finding has since been substantiated by other works [64]. Furthermore, there is evidence that the harmful effects of institutionalization are not limited to large institutions. Children in small-group care—such as SOS Children’s Villages—have substantial development delays as compared with their peers in family care [65, 66]. One respondent pointed to the strength of the scientific evidence:

The science is…pretty clear that you know, a child needs a family (I1).

Others expressed concerns about the lack of scientific rigor in research supporting claims that residential care is comparable to the care received in family-based arrangements (I23, I29):

[There is] dangerous research [conducted by a] minority that is academically questionable saying that the clean institutions and residential care and group homes are just as good as foster care…. There is a very serious misunderstanding of science out there…They are not paying attention to the greater amount of science that shows that residential care and group homes are dangerous, and the children need to be with families (I23).

Another DI argument is that institutionalization is not cost-effective and diverts resources from preventing family separation and strengthening families (I1, I12, I14). Research from the United States, for instance, indicates that group placements cost seven to ten times the cost of placing a child with a family [67]. One DI proponent made the additional point that:

The more we continue to pour resources into improving institutions, the less resources [there are] for helping families take care of their children (I7).

DI proponents also point to evidence on the heightened risk to institutionalized children of neglect and abuse from caregivers and peers [68–72]. For example, a Romanian study found that 38% of 7 to 18-year-olds in residential care reported severe punishments or beatings [73]. In addition, DI proponents point to the social and psychological harm, especially in terms of attachment, that institutionalized children experience given the constant overturn of volunteers in such facilities, and the involvement of untrained and non-certified caregivers [74].

In addition, DI proponents note that approximately 80–90% of the millions of children living in orphanages have at least one living parent [74, 75]. Accordingly, proponents argue that DI is critical to reintegrating these children back into their biological families, with support services to strengthen a family’s capacity to care [76].

#### Arguments by those who identify as DI-critical

Those who identity as DI-critical question the quality of the evidence that purports to show that all forms of care that are not family-based have more pronounced adverse effects on children than those that are family-based (I11, I14, I16, I19, I21, I25) [77]. They express concern (I10, I16, I18) [78] that analyses examining the effects of care arrangements that are not family-based on child well-being have focused largely on large hospital-style facilities caring for infants with shift workers, and that studies providing evidence on the harmful consequences of care arrangements that are not family-based are based predominantly on the experiences of states in the former Soviet bloc [79, 80]. Critics also express concern about design and methodological problems in studies of care arrangements that are not family-based [81]. One respondent noted his frustration with the body of literature that claims any care arrangement that is not family-based is universally detrimental:

Nobody seems ever to take account of the trauma [children have] been through before placement in care…The very fact they’re in care indicates that some degree of pre-care trauma has taken place (I27).

Critics also note evidence that family-based care can have adverse effects. A number of studies highlight that vulnerable and orphaned children are often exposed to significant levels of violence and abuse within extended-family care settings [82]. Critics note also that in some instances outcomes for children in care arrangements that are not family-based may be equal or better than those for their counterparts living in kinship care arrangements on outcomes pertaining to child rights, [83] nutritional status, [84] and mental health [85].

DI critics argue that more nuanced approaches tailored to context are needed (I10, I11, I14, I19, I25). They object to DI’s universal approach, concerned that it is too narrow a goal:

I really don’t think we can do black and white, especially when it comes to children. And if we do black and white that all children have to be in a family…then we’re not doing what the [UN Guidelines] say and the guidelines really ask us for an individualized approach for every child (I19).

Critics worry also about consequences for children in various care facilities when these are shut down (I9, I10, I11, I14, I15, I18, I25). There is concern that donor-led strategies have imposed unrealistic goals for DI implementation:

[There] has been a pressure on the countries to close institutions without having a clear roadmap for what should be there instead, and sometimes it’s done too hastily and without good, proper assessment for each individual child (I25).

Those concerned with the current DI approach also point to the fact that not all biological parents or extended families are capable of or have a desire to care for their children:

So you get other bold statements like: ‘A large majority of children living in institutions have one living parent or existing family.’ So what? That says nothing about the willingness, ability or capability of those people to be the caregiver. If they’re not willing, it doesn’t matter if they’re alive (I27).

Critics contend that more attention should be given to the “quality of care provided within a setting, whether that setting be family-based or institution-based,” rather than eliminating all forms of care that is not family-based [78].

### Factors shaping disagreements on deinstitutionalization as a strategy

Aside from differences on what DI actually entails, several additional factors have shaped care proponent disagreements on the legitimacy of DI as a strategy, and global priority for the issue of children’s care.

#### Data inadequacies

Insufficient data on the scope and nature of the problem have intensified proponent differences (I2-I4, I6, I8, I12-I14, I21, I26, I27):

How many [orphanages] are unlicensed? We don’t know. Number in children care? We don’t know. Number of economic orphans versus orphans? We don’t know (I11).

While efforts exist to improve data collection, [62, 64] difficulties persist in detecting the number of vulnerable and orphaned children. Divergent perspectives on the definition of ‘orphan’ and its usefulness as a designation shape these difficulties, [86] as does the dearth of accessible data from household surveys on children living outside of family care. With the exception of a few scattered estimates from a handful of countries and emergent efforts to better use household survey data, [87–89] vulnerable children are “largely falling off the statistical map” [90].

#### Contradictory evidence on solutions

A sometimes conflicting evidence base on solutions also leads to proponent differences. This is underpinned by the fact that robust impact measures for a number of areas of child well-being are not available, and ‘quality of care’ is a difficult concept to measure. Few longitudinal studies follow cohorts of children, especially in LMICs [59]. Those studies that do exist present contradictory results on the effects on children of small group homes versus other care arrangements [65, 66, 91, 92]. Yet another problem is that many of the studies examining care arrangements and interventions are based on experiences of former Soviet bloc countries [79, 80, 93]. One respondent noted:

You can’t prove the points to policymakers without the data (I27).

#### Divergent experiences between former Soviet bloc and other countries

Divergent experiences between former Soviet bloc and other countries have also fueled DI disagreements. Many with experience in the former tend to favor a strong DI approach given the history of child maltreatment in large-scale institutions in these countries. Many of those who support DI were moved by the horrifying images that surfaced in the 1990s of thousands of neglected children housed in overcrowded, state-run orphanages in Romania and other Eastern European countries (I9, I11, I18). One respondent notes that Lumos and Hope and Homes, for instance, both arose out of the Romanian experience (I18).

DI critics, in contrast, argue for a more nuanced approach given experiences with different child demographics and care arrangements that are more typical in the rest of the world. In low-income countries, children on average are considerably older than those of former Soviet bloc countries, as access to basic services and education drives children home placement. The majority of children living in alternative care across Sub-Saharan Africa are in family-based arrangements—living with a surviving parent, grandparent, or other family member [17, 94, 95].

Furthermore, there are differences in the way that existing care systems are organized. Highly centralized and government-regulated systems—which is more amenable to a DI approach—are common among post-Soviet countries. In contrast, decentralized and poorly regulated systems often characterize low-income countries, where private and often faith-based organizations dominate the landscape (I29, I30). One DI-critical respondent expressed frustration about the lack of recognition of the different realities:

The evidence that isn’t being absorbed by the DI community is that this whole DI movement started out as the former Soviet Union with large state-run institutions… But the profile of the child in care worldwide is very different than that reality (I18).

#### Challenges in introducing formal alternative care arrangements

The lack of social protection and legal systems and capacity to implement formal alternative care arrangements in many low-income settings lead some proponents to question the feasibility of DI as a strategy. While many countries have made efforts to establish legal and statutory frameworks for childcare reforms, the implementation of these laws has been challenging, given the lack of financial resources, inadequately qualified staff, and poor service provision. In Uruguay and Guatemala, police, rather than social workers, assess protection risks and make most referrals. Cultural, religious, and social resistance to certain alternative care arrangements also exist. In a number of countries alternatives such as domestic foster care or adoption are resisted because of unease with the idea of families raising another person’s child [23]. In most African countries, formal adoption is rare. One respondent describes the problem this way:

Foster care, development of small group homes, development of domestic adoption have all been proposed…we’re asking other countries to develop a whole type of profession …not necessarily something that naturally comes out of their own country environment. So, it can feel like an imposed solution (I8).

#### Commercial interests

The fact that individuals and organizations profit from institutional care and therefore have an interest in sustaining their existence also fuels disagreements on DI as a strategy. DI proponents argue that this phenomenon heightens the need for their closure:

If you don’t close the institutions, the places will be filled with new children. Without strong gatekeeping and actually closing them, you will have new children coming in (I25).

Others, while acknowledging these interests, believe that DI proponents use these as an excuse to promote an uncompromising position.

Few in the children’s care community deny that commercial interests shape the persistence of residential care (I4, I8, I25). Employees of residential facilities fear losing their jobs. Poor families, often seeking better education or health for their children, are sometimes pushed by orphanages engaged in intercountry adoption or residential facility managers driven by profit into giving up their children. Some parents do not understand the legal implications of adoption, signing papers without understanding that the provision is permanent [96]. Orphanage tourism—fueled by foreigner desires during their travels to help local children—motivates operators to set up new residential facilities and to use illicit practices to recruit clients [97–100]. Strong business interests make residential care facilities hard to close. Furthermore, government officials often support these facilities:

Government officials often have significant investments in orphanage care because it’s a lucrative business model for them (I18).

While acknowledging this reality, one respondent expressed frustration about how some DI proponents exaggerate the pernicious nature of profit-making in these facilities:

I’m not saying that never happens, but you cannot generalize globally that …the only reason why children are in child protection systems is because evil people who run orphanages want to make money (I15).

#### Perspectives of children, families and the disability community

Tensions about the acceptability of residential care have also been shaped by the perspectives of two historically under-represented groups in childcare reform debates: the children and families directly affected by care reform, and the disability community.

Children and young people themselves sometimes express a preference for residential over family-based care alternatives (I2, I11, I14, I19, I21, I28). This was found to be particularly true for teenagers who are at the stage where “the idea of having a family is not the most important thing” or for children who have already been through multiple foster placements that have failed (I2, I21). HIV positive children have also reported benefiting from important protective factors. These include a sense of belonging and appreciation for community, and gaining the resilience for coping with challenges such as stigma [101]. Practitioners working with young, unaccompanied migrants stress that:

It is really important to keep that menu of [care] options really open, be really conscious about what profiles may be more appropriate for certain types of care, really listening to the children themselves (I28).

On the other hand, most disability rights groups strongly support the DI agenda. They point to numerous declarations that advance the right of children with disabilities to live with their families, including the Convention on the Rights of Persons with Disabilities [102]. In 2017, the Committee on the Rights of Persons with Disabilities advanced the right of a child to grow up in a family, finding institutions—regardless of quality or size—to be unacceptable alternatives.

Disability Rights International (DRI) and partner organizations subsequently called for the UN General Assembly to include in its Resolution on the Rights of Children a recognition that there are “no exceptions to the right to grow up in a family for any child, and the provision of care never justifies the denial of this right”. While some care reform proponents sympathize with the concerns expressed by disability rights advocates, others express frustration with claims that residential care is akin to encroaching on a child’s human rights:

The problem is that they are mixing up dogmatic rights and justified claims…They’re saying basically that putting a child in a residential care home is tantamount to violating that child’s rights. And it’s not (I13).

### Consequences of problem definition disagreement

Difficulties with problem definition have hindered the ability of the children’s care community to address strategic challenges concerning governance and positioning.

#### Weak governance

Proponents identify numerous fault lines that hamper the establishment of effective governance arrangements for the children’s care community, ones that could enable actors in the sector to more effectively work together. The primary fault line is grounded in the problem definition difficulties pertaining to DI strategy. As one respondent puts it:

There [are] differences of opinion about what it is we’re arguing for and what the solutions are that we’re putting forward, which preoccupy us as a community…[and] stops us from being very cohesive (I8).

Proponents identify additional fault lines based on organizational focus and type; for example, between organizations largely active in and drawing on experiences in Eastern Europe and those focused on care reform in Sub-Saharan Africa and Asia (I9).

Proponents note that fragmentation is driven not just by differences over ideas, but also by turf. Respondents identify power struggles, with contention over control of the childcare reform agenda and desires to gain credit for contributions (I4, I5, I6, I7, I9, I11, I18, I21, I27). Respondents express concern over the uncomfortable environment in the children’s care sector that has emerged (I2, I5, I10, I11, I15, I25):

Organizations are trying to shut each other up rather than having a proper conversation (I2).

The ideology becomes a battering ram…If you’re not on board, you are an outcast. If you’re not singing from the hymnal, you’re excommunicated from the community (I20).

Proponents perceive the sector’s lack of resources to have contributed to competition (I2, I3, I5, I7, I12, I13, I27), resulting in organizations becoming “connected with their model” and unable to “step back and look at these issues free of their organizational needs” (I3). Effective leaders—individual and institutional—might help in transcending these challenges, but respondents note a dearth of unifying champions for childcare reform (I3, I6, I9, I13, I18).

Governance challenges include difficulties with coalition-building with the variety of sectors shaping children’s care, including education, child protection, health and justice (I2, I5, I8, 19, I14, I19, I24, I26, I28). One proponent expressed frustration that:

The very sectors and actors that need to be at the table to actually create the reforms we need aren’t there… And so it’s like we’ve got our eyes on the symptoms, not on the root causes (I5).

Some note disinterest or active resistance by these sectors in connecting their issues to children’s care because of resource constraints, bureaucratic red tape, or lack of awareness of how their issue relates.

The greatest lost opportunity proponents note has been the inability to capitalize on the recent surge in attention for addressing violence against children (VAC) (I2, I3, I5, I8, I9, I12, I14, I21, I25). Despite some efforts to advance linkages with the VAC sector, [103, 104] some children’s care proponents believed that they themselves were in part to blame for not collecting evidence to foster collaboration (I9). Others blame VAC sector actors for this disconnect, perceiving them to have “elbowed care issues out of the way” in their attempts to bring their issue prominence in the SDG discussions (I2).

#### Unconvincing positioning

Differences surrounding problem definition have made it difficult for care proponents to address the challenge of positioning: advancing a clear case that motivates policy-makers and civil society groups to act. Divergent approaches to childcare reform lead to policy-maker confusion concerning what they are being asked to do (I9, I18, I21, I29). Respondents note that care reform proponents lack a “collective elevator speech” (I18). Respondents express concern that the terminology actors in the sector use —such as institution and alternative care—is ambiguous and complex (I3, I9, I25).

As long as we can’t define what an institution is and what it looks like, it makes it hard to then advocate for governments not to support it (I9).

One of the difficulties with making the case for children’s care are misperceptions individuals have about orphanhood being the problem (I29) and the best way to support orphans. These misperceptions encourage the institutionalization of children and orphanage volunteerism. It is difficult to redirect an individual’s well-meaning support of orphanages via volunteering, donations, and faith-based mission work to support efforts to help strengthen and unify families. One respondent noted the difficulties in striking a balance in messaging that combats these public misperceptions, but that also does not deter the public from supporting children’s care all together:

How do we nuance the message so that you can say: ‘Yes eager person wanting to volunteer overseas, we still want you to maintain your optimism and go and learn something but at the same time we don’t want you to volunteer in an orphanage’…and the backlash of them thinking: ‘oh well I thought I was doing something good and now I’ve been told I’m doing something bad, so I don’t trust it at all and I don’t want to do anything’ (I9).

Another positioning concern, one even DI supporters acknowledge, is that they have placed too much emphasis on DI itself to the neglect of the role of family (I3, I6, I8, I18). The focus on institutions, the role of residential care, and DI in particular, are portrayed as the sector’s end goal, rather than as a “steppingstone” (I19), or one among multiple goals. Respondents say that in demanding the closing of institutions, proponents over-emphasize the problem and lack a positive messaging (I3, I11, I25).

Some also note their concern about how the current DI messaging hinders meaningful engagement of the most important stakeholders on this issue—affected children and parents—instead giving prominence to the roles of government officials and orphanage personnel in the childcare reform process:

Parents and children have been a real lacking group of people in this field…I’ve heard a lot about the governments and a lot about the orphanage leaders, but I haven’t heard a lot about the real agency in the room (I11).

Respondents noted how a focus on the family rather than discussions explicitly calling for DI are more likely to resonate with governments (I10, I11, I19):

[Care proponents were] having debates about small group homes and then you just have to give them a reality check and say: ‘Listen guys, this is not going to fly politically’.… Once you start talking about family, they’re more open to that, it’s less controversial (I19).

## Discussion

Given the scope of the problem, global priority for children’s care remains insufficient. While multiple factors hinder priority generation—many not directly under the control of the global actors concerned with the issue—one factor connected to this set of actors may be influential: problem definition disagreements pertaining to the acceptability of care arrangements that are not family-based, and DI as a care reform strategy. Multiple factors have shaped these disagreements, including a contradictory evidence base on the scope of the problem and solutions, divergent experiences between former Soviet bloc and other countries, socio-cultural and legal challenges in introducing formal alternative care arrangements, commercial interests that perpetuate support for residential facilities, as well as the sometimes conflicting views of impacted children, families, and the disability community. These problem definition disagreements contribute to difficulties on governance—establishing global institutions to facilitate collective action—and positioning—framing the issue to attract the support of policy-makers and civil society organizations. In line with agenda setting scholarship, these problem definition disagreements, ineffective governance, and unconvincing positioning of the problem may have hampered the children's care policy community from advancing greater global attention to this problem.

Despite these challenges, several developments portend well for priority generation. Multiple forums and networks aimed at bringing champions together are in place, including the Elevate Children Funders Children’s Care Working Group, the Better Care Network, the Coalition on Children Without Parental Care, and the Global Collaborative Platform on Transforming Children’s Care. Also, in 2019, the United Nations General Assembly selected ‘Children without Parental Care’ as the theme for the ‘Rights of the Child’ resolution. The latter catalyzed unprecedented unity among care sector proponents, as reflected by the agreement of hundreds of care sector actors on a set of key recommendations for the UNGA resolution that address key challenges and opportunities in implementing the rights of children without parental care. Furthermore, as a response to the COVID-19 pandemic, a number of countries advanced the return of children to families or family-based care to reduce the number of children in institutional care, and there has been some evidence of proponents momentarily putting aside their differences to address imminent and critical needs. Finally, data are growing on which interventions are most effective to support children in families, prevent unnecessary separation, and provide quality alternative care when they cannot live with their parents or families. By overcoming existing challenges and capitalizing on opportunities, proponents concerned with child care and social work will be in a better position to advance policy attention to their issue, attract more donor and government funding, and ultimately improve the well-being and care of orphans and vulnerable children across the world.

### Strategic considerations for advancing global priority for children’s care

Advancing global priority for children’s care may require proponents to address the impasses that exist within their community. Proponents are unified by a deep concern for the well-being of children who lack adequate care. But they are divided deeply by disagreements on how best to address this problem, resulting in a number of acrimonious relationships among policy community members. The situation inside the community has features of a hurting stalemate, with entrenched policy positions. Needed are venues to bring actors together that enable them to stand back from certain beliefs and consider the policy positions of those with whom they disagree: for DI supporters to consider the limits of the current strategy and for DI critics to consider its merits. Immediate policy windows and crises may provide incentives to do so. This may include building on the relative attention now being given to preventing violence against children, the imperative to address present children’s care crises (i.e., pertaining to Afghani and Ukrainian refugees and Central American migrants to the United States), and the global impacts of COVID-19 on vulnerable families and children.

Without transcending problem definition difficulties, it will likely be difficult for proponents to manage other strategic challenges, including developing a convincing positioning that motivates policy-makers and donors to act. In particular, proponents will need to consider how focus on the issue of childcare reform may have crowded out attention to other critical components of the children’s care agenda such as family strengthening and the potential value of frames that better encompass the wider agenda. Also, proponents will need to consider how to surmount ambiguity surrounding terms such as institutional care, residential care, and alternative care, communicating in ways that key audiences understand. In addition, proponents will need to specify what exactly they are asking of policy-makers and of well-meaning individuals who want to support vulnerable and orphaned children, identifying simple, positive actions. Better positioning strategies will also help with coalition-building—creating alliances with other sectors necessary for advancing the children’s care agenda. Care proponents will need to strategize about how to gain seats at the table of entities that exercise leadership in other sectors—such as the Global Partnership to End Violence Against Children and the Global Partnership for Education—to ensure these and other forums actively consider concerns related to children’s care. Finally, while some efforts of this kind are emergent, [105] care proponents should explore avenues to pursue more active engagement with the disability community, as well as families and children directly affected. Not only will such engagement increase the care sector’s legitimacy and improve its visibility, doing so will also expand the set of grassroots allies that can advance children’s care.

This study has several limitations. First, we recognize the bias that may be introduced in our flexible approach to sourcing the documents, as this may have led to us missing critical documents or over-sampling documents advancing a particular perspective. Relatedly, our approach to sourcing the documents and selecting key informants is not entirely reproducible. However, a strength of our search and selection strategies is that they evolved as we incorporated new data and suggestions from key informants. Furthermore, any biases introduced during the data collection, analysis, and manuscript writing phases of this research were minimized given our positionality (researchers outside of the children’s care sector with no strong views about alternative care reform or the DI debate); our purposeful sampling strategy (seeking to identify individuals holding different perspectives and positions, i.e., asking key informants to identify individuals in the sector with similar and different perspectives from them on childcare reform and purposefully seeking out a balance of individuals with distinct perspectives that work in donor agencies, NGOs, and international institutions with various mandates and geographic focuses); our triangulation of multiple data points (i.e., between key informant interview and literature data); and our sharing a draft of the analysis with four experts on the subject—representing the distinct perspectives we identified—to check the accuracy and balance of the perspectives presented.

A second limitation is that only one individual undertook the data coding. Accordingly, formal interrater agreement was not established which may skew the interpretation and presentation of the data, although both authors discussed emergent themes and how to organize the data throughout the data collection, analysis, and manuscript writing phases. In an effort to validate the findings and present the perspectives identified in a balanced way, the authors were intentional in including the key points of reasoning advanced by each perspective, reserved similar space (words, citations, quotes) for discussing the rationales of each of the distinct perspectives, and incorporated feedback on a draft of this paper from four interviewees representing the distinct perspectives identified.

A third limitation is our decision to draw on particular agenda setting scholarship and frameworks, which shapes the conduct and presentation of our analysis. However, we tried to account for this in our approach by not only deductively coding our data in line with theoretical scholarship, but also incorporating an inductive approach to account for emergent themes that were not outlined by our original codes. Finally, this study was conducted at the global level, and therefore the findings are not generalizable for explaining national-level prioritization of children’s care, which would need to account for country and region-specific developments, structures, and capacities. Nevertheless, some of the findings are likely transferable—such as the way in which tensions over the meaning of DI and its legitimacy may limit prioritization of children’s care by local policymakers and donors. This study not only provides hypotheses for further historical scrutiny and empirical testing, it offers a basis for further in-depth investigation on national-level agenda setting concerning children’s care. Future studies should consider more and less successful cases of country prioritization of addressing children’ care, specifically probing the strategies that proponents employ, as well as the contexts in which they operate. Analyses that draw on the experiences of countries with various contexts and levels of political priority for addressing children’s care will offer more generalizable insights for proponents across the globe.

## Conclusions

In summary, challenges surrounding problem definition are considerable within the children’s care community. These tensions are apparent at the global level among experts working in international organizations, donor agencies, and non-governmental agencies across countries; they also likely manifest within national and local contexts, impacting priority of children’s care at those levels as well. However, proponents have many advantages, not least of which is a shared concern over the well-being of children at risk. Given this commonality, there is no reason to believe that this community cannot find ways to transcend differences and potentially become a powerful agent of change to advance the children’s care agenda.

### Supplementary Information

**Additional file 1: Annex 1.** Key informant interview guided template.

## Data Availability

The dataset generated and/or analyzed during the current study are not publicly available given that individual privacy could be compromised but can be made available with significant retractions from the corresponding author on reasonable request.
