# Supplementary material for: Global priority for the care of orphans and other vulnerable children: transcending problem definition challenges
Source: Global Health. 2023 Oct 10;19:75. doi: 10.1186/s12992-023-00975-0 (PMC10566118; doi:10.1186/s12992-023-00975-0)
Supplement: Supplementary file 1 — Additional file 1: Annex 1. Key informant interview guided template. [file 12992_2023_975_MOESM1_ESM.docx]

**Annex 1: Key Informant Interview Guided Template**

**Level of prioritization**

1. To what extent do you believe that addressing children’s care is a priority on the global agenda? What evidence would you cite to support this?

**Governance and coalition-building**

1. Can you tell me about X [organization]’s role in addressing children’s care?
2. Which individuals and organizations comprise the child protection policy community and those specifically concerned with children’s care? To what extent do they constitute a cohesive policy community?
3. Are there individual champions/leaders that have been able to unify advocates, leading the charge for addressing children’s care?
4. What global guiding institutions or networks exist for coordinating collective action to advance addressing children’s care? How effective have these institutions/networks been?
5. To what extent do those concerned with child’s care constitute a cohesive policy community?
   1. In your opinion, does their lack of cohesiveness hinder their advocacy capacity?
6. What are the key points of agreement and disagreement among advocates concerned with children’s care? How well have proponents been able to manage and transcend differences?
7. What effects have these differences had on the community’s capacity to draw attention to the issues that concern them?

**Positioning**

1. How have proponents made the case to leaders of international institutions?
2. How well have proponents conveyed the severity of the problem?
3. How convincing are the solutions that proponents have proposed, and how effectively have they communicated solutions as a means of attracting political support?

**Issue characteristics**

1. What other features of the problem (i.e., sympathy for orphans) facilitate attention to the issue?
2. What dimensions of the problem (i.e., difficulty in accounting for children living outside of family care) make it more difficult to generate action?

**Policy environment**

1. Aside from those noted above, what other factors connected to political context have facilitated or hampered attention to children’s care—such as seminal studies, new intervention alternatives, and social movements?
2. What are the global accountability mechanisms for ensuring children’s care is appropriately addressed? Who is responsible for compliance?
